# RE-CONCEPTUALIZING SPOUSAL CAREGIVING FROM A DYADIC PERSPECTIVE IN THE U.S.

**DOI:** 10.1093/geroni/igz038.917

**Published:** 2019-11-08

**Authors:** Yuekang Li, Huiying Liu, Yi Wang, Nancy Morrow-Howell

**Affiliations:** 1 Washington University in St.Louis, St.Louis, Missouri, United States; 2 The university of Hong Kong, Hong Kong, Not Applicable, Hong Kong

## Abstract

Existing literature on later-life spousal caregiving tends to focus on one member of the marital dyads, assuming a priori distinction between the caregiver and care-receiver. Theoretically and empirically, this individualistic role-related (caregiver-receiver) approach is inaccurate, as the concept of spousal care intrinsically involves two people within a marital dyad. Therefore, this paper used a social exchange perspective to re-conceptualize spousal caregiving as a dyad-level phenomenon. Using the 2014 wave of the Health and Retirement Study, 6,500 individuals (3,250 couples) aged 65 and above and their spouse were selected. Based on each partner’s need for care, receipt of spousal care, and provision of spousal care, the study identified five distinctive caregiving typologies. Household-level factors such as the availability of other types of informal care were associated with these typologies. This paper offers a broader and more dynamic perspective of the spousal caregiving experiences.

